# Pancreatic steatosis and iron overload increases cardiovascular risk in non-alcoholic fatty liver disease

**DOI:** 10.3389/fendo.2023.1213441

**Published:** 2023-08-03

**Authors:** David Marti-Aguado, Amadeo Ten-Esteve, Carlos Manuel Baracaldo-Silva, Ana Crespo, Elena Coello, Víctor Merino-Murgui, Matias Fernandez-Paton, Clara Alfaro-Cervello, Alba Sánchez-Martín, Mónica Bauza, Ana Jimenez-Pastor, Alexandre Perez-Girbes, Salvador Benlloch, Judith Pérez-Rojas, Víctor Puglia, Antonio Ferrández, Victoria Aguilera, Mercedes Latorre, Cristina Monton, Desamparados Escudero-García, Ignacio Bosch-Roig, Ángel Alberich-Bayarri, Luis Marti-Bonmati

**Affiliations:** ^1^ Digestive Disease Department, Clinic University Hospital, INCLIVA Health Research Institute, Valencia, Spain; ^2^ Biomedical Imaging Research Group (GIBI230), La Fe Health Research Institute, and Imaging La Fe node at Distributed Network for Biomedical Imaging (ReDIB) Unique Scientific and Technical Infrastructures (ICTS), Valencia, Spain; ^3^ Department of Technologies for Health and Well-Being, Polytechnic University of Valencia, Valencia, Spain; ^4^ Radiology Department, La Fe University and Polytechnic Hospital, Valencia, Spain; ^5^ Digestive Disease Department, Hospital Arnau de Vilanova, Valencia, Spain; ^6^ Hepatology and Liver Transplantation Unit, La Fe University and Polytechnic Hospital, Valencia, Spain; ^7^ Pathology Department, Clinic University Hospital, INCLIVA Health Research Institute, Valencia, Spain; ^8^ Faculty of Medicine, University of Valencia, Valencia, Spain; ^9^ Pathology Department, La Fe University and Polytechnic Hospital, Valencia, Spain; ^10^ Quantitative Imaging Biomarkers in Medicine, QUIBIM SL, Valencia, Spain; ^11^ CIBERehd, Centro de Investigación Biomédica en Red en Enfermedades Hepáticas y Digestivas, Instituto de Salud Carlos III, Madrid, Spain; ^12^ Pathology Department, Hospital Arnau de Vilanova, Valencia, Spain; ^13^ Hepatology Unit, Consorcio Hospital General Universitario de Valencia, Valencia, Spain; ^14^ Universitat Politècnica de València, Institute of Telecommunications and Multimedia Applications (iTEAM), Valencia, Spain

**Keywords:** non-alcoholic fatty liver disease (NAFLD), magnetic resonance imaging (MRI), proton density fat fraction (PDFF), pancreatic steatosis, iron overload, cardiovascular risk

## Abstract

**Objective:**

To assess the prevalence of pancreatic steatosis and iron overload in non-alcoholic fatty liver disease (NAFLD) and their correlation with liver histology severity and the risk of cardiometabolic diseases.

**Method:**

A prospective, multicenter study including NAFLD patients with biopsy and paired Magnetic Resonance Imaging (MRI) was performed. Liver biopsies were evaluated according to NASH Clinical Research Network, hepatic iron storages were scored, and digital pathology quantified the tissue proportionate areas of fat and iron. MRI-biomarkers of fat fraction (PDFF) and iron accumulation (R2*) were obtained from the liver and pancreas. Different metabolic traits were evaluated, cardiovascular disease (CVD) risk was estimated with the atherosclerotic CVD score, and the severity of iron metabolism alteration was determined by grading metabolic hiperferritinemia (MHF). Associations between CVD, histology and MRI were investigated.

**Results:**

In total, 324 patients were included. MRI-determined pancreatic iron overload and moderate-to severe steatosis were present in 45% and 25%, respectively. Liver and pancreatic MRI-biomarkers showed a weak correlation (r=0.32 for PDFF, r=0.17 for R2*). Pancreatic PDFF increased with hepatic histologic steatosis grades and NASH diagnosis (*p*<0.001). Prevalence of pancreatic steatosis and iron overload increased with the number of metabolic traits (*p*<0.001). Liver R2* significantly correlated with MHF (AUC=0.77 [0.72-0.82]). MRI-determined pancreatic steatosis (OR=3.15 [1.63-6.09]), and iron overload (OR=2.39 [1.32-4.37]) were independently associated with high-risk CVD. Histologic diagnosis of NASH and advanced fibrosis were also associated with high-risk CVD.

**Conclusion:**

Pancreatic steatosis and iron overload could be of utility in clinical decision-making and prognostication of NAFLD.

## 1 Introduction

Non-alcoholic fatty liver disease (NAFLD) is the most common chronic liver disorder and is associated with insulin resistance and increased risk of cardiovascular disease (CVD) (1). NAFLD is also an important contributor to morbidity in other organs beyond the liver, as determined by increased incidence of extrahepatic diseases such as type-2 diabetes mellitus (DM) and arterial hypertension (AHT) (2). In this context, CVD is the leading cause of mortality in patients with NAFLD (3).

Magnetic Resonance Imaging proton density fat fraction (MRI-PDFF) is the most accurate non-invasive method for assessing liver steatosis (4). Beyond fat quantification, MR can simultaneously measure R2* which is a surrogated biomarker of iron concentration (5). These parameters have become widely available for the study of chronic liver diseases. Liver MR protocols include other organs within the acquisition volume, such as the pancreas. Recognizing the presence and distribution of disease in other organs is clinically relevant in a multisystemic disease such as NAFLD, which is associated with multiple metabolic disorders (3). This approach of looking outside the liver box can help to understand NAFLD clinical heterogeneity.

Pancreatic steatosis and iron overload are emerging clinical entities not as well characterized as NAFLD (6). In general population, the prevalence of pancreatic steatosis is 33%, and it is associated with AHT, DM, metabolic syndrome, and NAFLD (7, 8). Metabolic hiperferritinemia (MHF) is a common finding in NAFLD that reflects iron metabolism alteration that facilitate iron accumulation in different organs and is associated with metabolic disfunction (9). MRI is the most developed method to quantify pancreatic fat and iron storages (10). In NAFLD, contrasting results have been shown connecting liver fat content, NASH severity, pancreatic steatosis, and cardiometabolic risk (**Supplementary Material Table 1**). Disparities between studies can be due to limited sample sizes and methodological issues as most of them are single center. Furthermore, validated criteria for the non-invasive diagnosis of MHF and the staging of iron overload are still lacking and represent a research opportunity (9). In view of these knowledge gaps, additional prospective multicenter studies are required to investigate the relationship between liver and pancreas fat and iron accumulation, aiming to define the impact of this relationship regarding cardiometabolic disorders (8–10).

The primary objective of the current study was to investigate the prevalence of pancreatic steatosis and iron overload in a well-characterized cohort of biopsy proven NAFLD and to determine their association with liver histology and cardiometabolic conditions.

## 2 Materials and methods

### 2.1 Study design and population

This is a prospective, cross-sectional, multicenter study. Patients with NAFLD diagnosis and a clinical indication for liver biopsy were recruited at four medical centers (Valencia, Spain) between 2017-2022. Consecutive NAFLD patients were included based on increased liver enzymes and evidence of hepatic steatosis on ultrasound, in addition to either obesity, DM or metabolic dysregulation (11). Participants were invited to undergo a research MRI examination with a per-protocol time interval less than 30 days from biopsy. The participant inclusion criteria were age ≥18 years old and having signed the informed consent. Exclusion criteria were evidence of liver disease other than NAFLD, alcohol consumption (defined as daily alcohol consumption >20 g in women and >30 g in men), secondary causes of hepatic steatosis, contraindications to MRI, imaging artifacts, unsatisfactory biopsy sample, and hepatic or extra-hepatic malignancy. The study conforms to the ethical guidelines of the 1975 Declaration of Helsinki and had the approval of the institutional review boards of the participating hospitals.

### 2.2 Baseline characteristics and definitions

All participants underwent a standardized clinical evaluation at baseline, including age, sex, body mass index (BMI, kg/m^2^), waist circumference, metabolic comorbidities (obesity, DM, ATH, dyslipidemia) and metabolic syndrome diagnosis based on the Adult Treatment Panel III criteria (12). Obesity was defined as individuals with BMI ≥30 kg/m2. DM was defined by a fasting glucose level ≥126 mg/dL, self‐reported medical history of diabetes, oral hypoglycemic agents, insulin use, or HbA1c ≥6.5%. ATH was defined by a systolic blood pressure measure ≥130 mm Hg or diastolic blood pressure measurement ≥80 mm Hg from an average of three measurements or history of high blood pressure measurements. Dyslipidemia was defined as fasting plasma cholesterol >220 mg/dL, low-density lipoprotein (LDL) >130 mg/dL or being under lipid-lowering drugs. Laboratory parameters including liver, glycemic, lipid and iron complete panel were also collected. Fasting glucose and glycosylated hemoglobin (HbA1c) were measured as surrogated markers of insulin resistance. The visceral adiposity index (VAI) was calculated as a marker of adipose tissue dysfunction and metabolic risk. Diagnosis and severity of MHF was based on ferritin thresholds (9). Accordingly, serum levels of ferritin between 200 in women and 300 in men up to 550 ng/mL defined MHF, values between 550-1000 ng/mL corresponded to dysmetabolic iron accumulation and >1000 ng/mL established the diagnosis of dysmetabolic iron overload syndrome (9). The CVD risk was estimated with the Atherosclerotic Cardiovascular Disease (ASCVD) score that estimates the 10-year risk of coronary heart disease (CHD) (13). The ASCVD risk score was stratified according to American College of Cardiology/American Heart Association (ACC/AHA) guidelines: low (0% - 4.9%), borderline (5% - 7.4%), intermediate (7.5% - 20%) and high (>20%) (13). In this study, individuals with a 10‐year ASCVD risk score of ≥7.5% were referred to as high risk for CVD (14).

### 2.3 MRI acquisition and analysis

MRI (3T-TX Achieva, Philips Healthcare) were obtained with a sixteen-channel phased-array coil. Participants were asked to fast for a minimum of 4 hours. All participants had a standard non-enhanced MRI reviewed by a radiologist (A.P.G, 10 years of experience on abdominal imaging) to exclude focal liver and pancreatic abnormalities before image analysis. A 2D multiecho chemical shift-encoded gradient echo sequence was obtained in a single breath-hold acquisition with 12 echoes (TEs=0.9-7.9, short echo spacing=0.7 ms; TR=9 ms) and low flip angle (10°) to minimize T1 bias. Image postprocessing was performed with a fitting algorithm that corrects T2* effects and the spectral complexity of the fat signal (6 peak multifrequency) to calculate fat and iron contents (5).

Hepatic PDFF and iron-related R2* values were measured after automatic whole-liver segmentation (5). Based on a recent meta-analysis, we used an MRI-PDFF cut-off of 5.5% for the definition of any degree of steatosis and of 15.5% to identify moderate-to severe liver steatosis (15). All included participants had a liver PDFF ≥5.5%. Hepatic R2* cut-off value of 70 s^-1^ was used as the reference for increased hepatic iron and dysmetabolic iron accumulation (9, 16). Intrapancreatic PDFF and R2* were obtained with manual delimitation by a single experienced radiologist (CM.B.S), placing three regions of interest (ROIs) set to 50 mm^2^ were drawn on the head, body and tail of the pancreas avoiding the pancreatic duct, major vessel, adjacent visceral fat, and artifacts (open-source software ITK-SNAP v.3.6.0; http://www.itksnap.org) (17). The mean signal intensities from the three ROIs were employed to determine the average pancreatic fat fraction and iron overload. As recommended, pancreatic PDFF cut-off of 6.2% was used to define fatty pancreas, and of 15.5% to identify moderate-to severe steatosis (7, 15). A pancreatic R2* value above 39 s^−1^ was used to define pancreatic iron overload (16). Image analysts were blinded to clinical and histological data at the time of image analysis.

### 2.4 Histological evaluation

Percutaneous biopsies of the liver were obtained with a semiautomatic 16G two-step needle. After formalin fixation (10% buffered), paraffin-embedded tissue sections (4-µm thick) were stained with hematoxylin and eosin (H&E), PicroSirius red (0.1%, MERCK) for fibrosis detection, adipophilin immunohistochemistry (VITRO Master Diagnostica) for steatosis detection and Perls staining (Artisan Iron Staining Kit, DAKO) for iron assessment. All biopsies were centrally evaluated by experienced liver pathologists blinded to clinical and imaging data (C.A.C, A.S.M, and A.F.). Histological scoring used the Nonalcoholic Steatohepatitis (NASH) Clinical Research Network system to grade steatosis from S0-S3 and fibrosis from stage F0-F1 (18). Advanced fibrosis was defined as F3-4. Diagnosis of NASH was based on the presence of steatosis, hepatocyte ballooning and lobular inflammation. The grading of iron storage was assessed using Scheuer’s scoring system (Fe0-Fe4) (19). Additionally, all stained biopsies were digitalized with a Ventana iScan HT slide scanner (Roche, Ventana Medical Systems, Inc), capturing whole-slide digital images with a 40× magnification objective and a calibrated camera (4000 × 4000 pixels being 1 mm^2^). Then, digital image analysis was performed to quantitatively obtain the proportionate area (%) of fat and iron with a computerized algorithm based on enhanced color and shape-based thresholds (MATLAB, MathWorks, version R2016a) (20). No biopsy samples were obtained from the pancreas for histological evaluation.

### 2.5 Statistical analysis

Categorical data are expressed as frequencies (%), and quantitative data are expressed as the mean and standard deviation (SD) or median and interquartile range (IQR). Comparisons between histological grades in terms of MRI quantitative data were performed using the Kruskal-Wallis *post hoc* Tukey’s range test. Linear regression analysis was performed to determine the correlation (Spearman correlation coefficient [r]) between MRI-derived values and digital pathology data. Strength of correlation was interpreted according to 0.20-0.39 weak; 0.40-0.59 moderate; 0.60-0.79 strong; and 0.80-1.0 very strong (21). For precision repeatability evaluation, intraclass correlation coefficient (ICC) with 95% confidence intervals (CI) was calculated for pancreatic MRI-PDFF intra- and inter-measurement concordance. Paired t-tests were used to compare MRI-PDFF across different regions of the pancreas. Differences between cardiometabolic disorders and MRI biomarkers were evaluated using Mann-Whitney U test for continuous data and the chi-square or Fisher test for categorical data, as required. Due to few cases with ferritin levels >1000 ng/mL (n=9), the spectrum of iron metabolism was categorized as normal (ferritin ≤200 ng/mL in women and ≤300 ng/mL in men), MHF (ferritin >200/300 in women/men up to 550 ng/mL) and dysmetabolic iron accumulation (ferritin >550 ng/mL). Differences of R2* values between groups of iron metabolism were assessed with one-way analysis of variance ANOVA, with *post hoc* Bonferroni test. The receiver operating characteristic (ROC) curve and area under the ROC curve (AUC) were applied to establish the diagnostic accuracy of R2* to detect iron metabolism alteration. The ASCVD risk score was dichotomized as low (<7.5%) and high (≥7.5%) (14). Baseline characteristics that could have an influence on CVD risk, such as gender, age, tobacco consumption, and dyslipidemia were included in the logistic regression model, to express adjusted odds ratio (OR) with 95% confidence interval (CI). A *p*-value <0.05 was considered statistically significant. Analyses were performed with the SPSS V25.0 software package.

## 3 Results

### 3.1 Baseline characteristics

**Figure 1:**
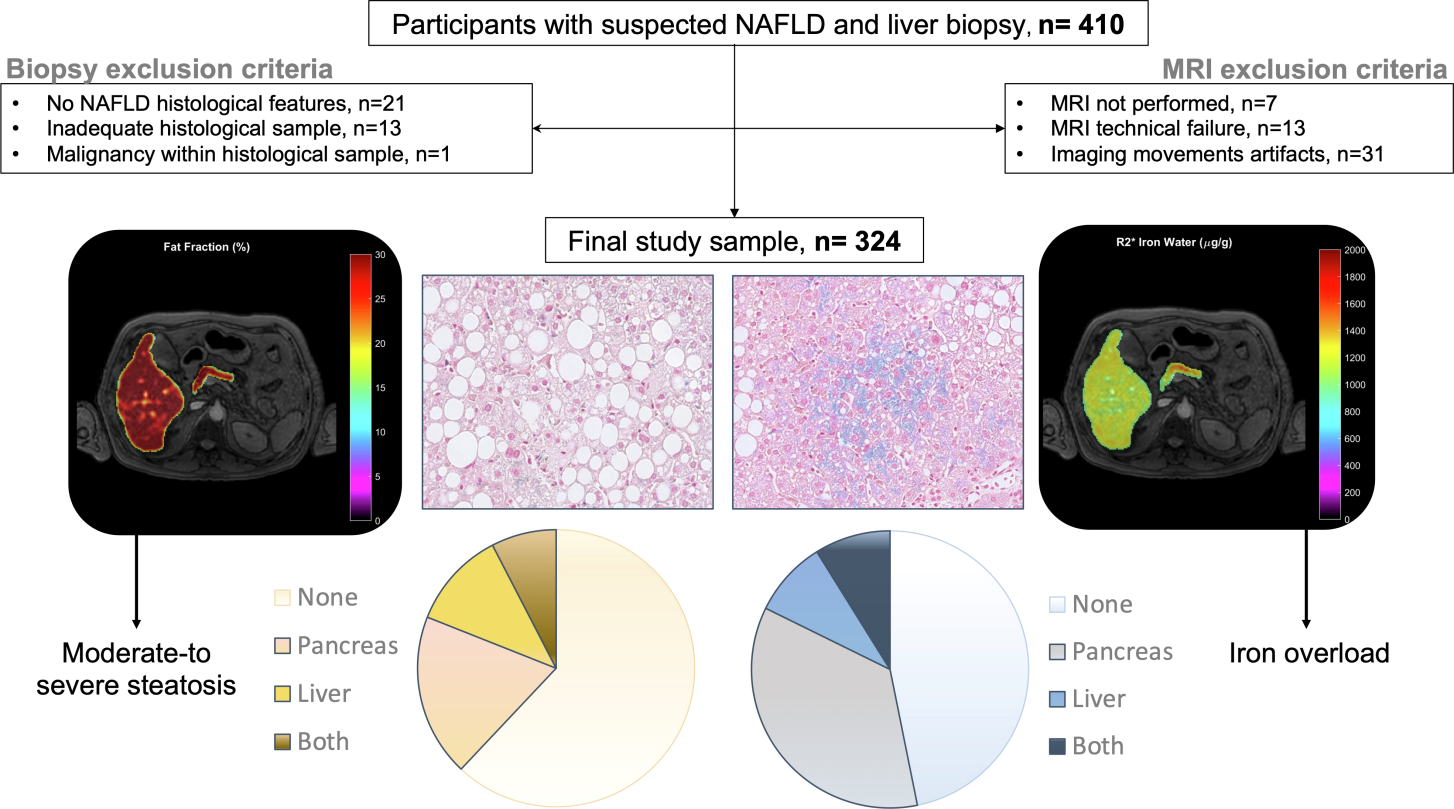
Participant flowchart, schematic overview of diagnostic techniques and organ distribution of MRI assessed steatosis and iron overload.

In total, 324 patients were included (**Figure 1**). **Table 1** summarizes patients´ clinical and biochemical characteristics. The study cohort had 60% (n=194) females with a mean age and BMI of 55 years ( ± 11) and 28.2 kg/m^2^ ( ± 5.1), respectively. The distribution of iron metabolism and CVD risk included 13.6% (n=42) with MHF, 7.4% (n=23) with dysmetabolic iron accumulation, and 37.1% (n=117) with high CVD risk. Histological review showed that 38.6% (n=125) were classified as NASH, 18.8% (n=54) had increased iron deposits and 24.1% (n=78) presented advanced fibrosis (**Supplementary Material Table 2**). The median time interval (IQR) between biopsy and MRI was 19 (14-27) days.

**Table 1:** Baseline clinical and laboratory characteristics.

Characteristics	Patients
Female sex	194 (59.9%)
Age (years)	55 ± 11
BMI (kg/m^2^)	28.2 ± 5.1
Mean arterial pressure (mmHg)	92 (83 – 99)
Tobacco consumption (ever smoker)	78 (24.0%)
Metabolic traits:	
•Metabolic syndrome	97 (29.9%)
•Hypertension	129 (39.8%)
•Diabetes Mellitus	94 (29.0%)
•Dyslipidemia	170 (52.5%)
Waist circumference (cm)	98 ± 17
Visceral adiposity index (VAI)	1.32 (0.83 – 2.23)
Iron metabolism:	
•Metabolic syndrome	244 (79.0%)
•Metabolic hiperferritinemia	42 (13.6%)
•Dysmetabolic iron accumulation	23 (7.4%)
ASCVD risk score	
•Low (0 - 7.4%)	198 (62.9%)
•High (≥ 7.5%)	117 (37.1%)
Platelet count (×10^9^/L)	228 ± 79
Creatinine (mg/dL)	0.8 ± 0.2
Glucose (mg/dL)	108 ± 36
HbA1c (%)	6.1 ± 1.2
ALT (U/L)	51 (34 - 82)
AST (U/L)	41 (31 - 65)
GGT (U/L)	83 (46 - 179)
Total bilirubin (mg/dL)	0.6 (0.5 – 0.8)
Ferritin (ng/mL)	105 (44 - 230)
Albumin (g/dL)	4.4 (4.2 - 4.6)
Triglycerides (mg/dL)	111 (77 - 170)
Total cholesterol (mg/dL)	191 ± 43
Low-density lipoprotein (mg/dL)	115 ± 36
High-density lipoprotein (mg/dL)	53 (44 - 68)

Data is expressed as numbers of participants, with percentages in parentheses or means ± SD when normally distributed and medians with IQR when the distribution is skewed.

ALT, alanine aminotransferase; ASCVD, atherosclerotic cardiovascular risk; AST, aspartate aminotransferase; BMI, body mass index, GGT, g-glutamyl transferase; HbA1c, glycosylated hemoglobin.

### 3.2 Pancreatic and liver steatosis

**Figure 2:**
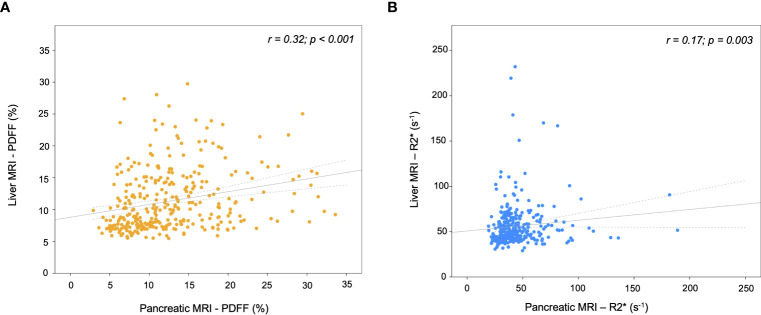
Scatterplots of MRI parameters in the liver versus pancreas. **(A)** Proton density fat fraction (PDFF), and **(B)** transverse relaxometry (R2*). The gray line represents the linear regression fit and the dotted lines the 95% confidence interval.

**Figure 3:**
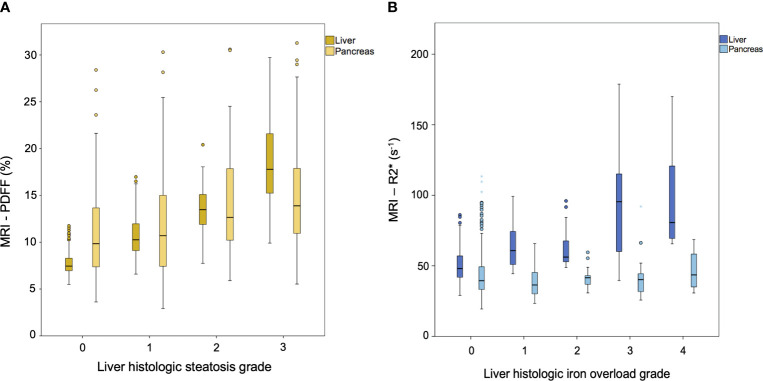
Box and whisker plots of MRI markers of **(A)** proton density fat fraction (PDFF, expressed as percentages) and **(B)** transverse relaxometry (R2*, expressed as s^-1^) versus histologic grades. **(A)** Dark-yellow boxes correspond to liver PDFF and light-yellow boxes to pancreas PDFF. **(B)** Dark-blue boxes correspond to liver R2* and light-blue boxes to pancreas R2*.

**Figure 4:**
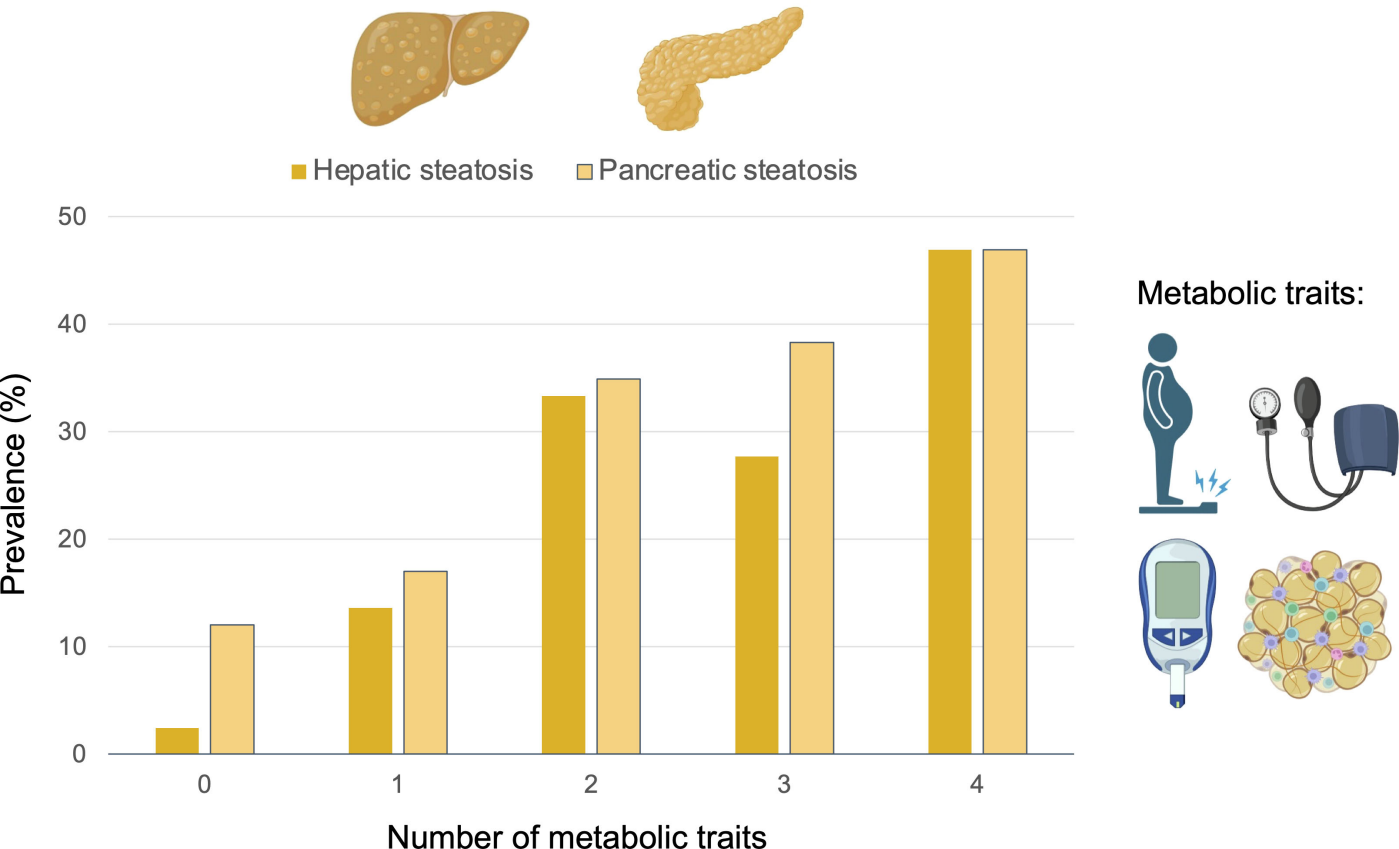
Prevalence (%) of moderate-to severe hepatic and pancreatic steatosis depending on the number metabolic risk factors. The metabolic traits considered were obesity, arterial hypertension, type-2 diabetes mellitus, and dyslipidemia.

Mean pancreatic and liver MRI-PDFF were 12.6 ± 6.0% and 11.3 ± 4.8%, respectively. The mean pancreatic fat content did not vary significantly between the head, body, and tail of the pancreas (**Supplementary Material Figure 1**). Pancreatic PDFF intra- and inter-measurement ICC was 0.75 (95%CI 0.68-0.81) and 0.88 (95%CI 0.85-0.90), respectively. The prevalence of pancreatic steatosis and moderate-to severe fatty pancreas was 90% (n=292) and 25% (n=82), respectively. No differences were observed between gender (*p*=0.970). The prevalence of moderate-to severe steatosis in the liver was 19.8% (n=64) and in both pancreas and liver was 8.3% (**Figure 1**). There was a weak correlation between pancreatic PDFF and both liver PDFF (r=0.32, *p*<0.001; **Figure 2**) and digital pathology fat proportionate area (r=0.29, *p*<0.001; **Supplementary Material Figure 2**). Pancreatic PDFF values increased significantly with hepatic histologic steatosis grades (**Figure 3**). Pancreatic PDFF was associated with NASH diagnosis (mean PDFF 11.7% in non-NASH *vs*. 14.5% in NASH, *p*<0.001), but no correlation was found with liver fibrosis stages (**Table 2**). Moderate-to severe fatty pancreas was associated to all metabolic disorders, showing an exponential increase with the number of metabolic traits (**Figure 4**). The mean pancreas MRI-PDFF was significantly higher in patients with DM compared with non-diabetic patients (14.6 ± 7.0% *vs*. 11.9 ± 5.3%, p<0.001). DM was associated with both pancreatic (OR: 2.23, 95%CI 1.31-3.82) and liver (OR: 2.72, 95%CI 1.53-4.82) moderate-to severe steatosis. In non-diabetic patients, fasting glucose and HbA1c significantly increased with both pancreatic and liver moderate-to severe steatosis (**Supplementary Material Table 3**).Overall, liver fat content but not pancreatic steatosis correlated with VAI (r=0.05 for pancreas, and r=0.34 for liver) and BMI (r=0.27 for pancreas, and r=0.54 for liver).

**Table 2:** Distribution of pancreatic MRI-metrics across liver histological grades.

Liver histological features	Pancreatic PDFF (%)	*p* value	Pancreatic R2* (s^-1^)	*p* value
Steatosis grade		<0.001		0.114
•S0	9.8 (7.3-13.6)		36.9 (31.5-45.9)	
•S1	10.7 (7.5-15.0)		37.8 (32.9-47.3)	
•S2	12.6 (9.6-18.1)		39.7 (33.9-47.8)	
•S3	13.9 (10.9-18.1)		43.5 (35.5-57.6)	
Lob. inflammation		0.101		0.277
•I0	10.8 (8.3-15.2)		36.8 (31.1-47.4)	
•I1	12.2 (8.5-15.6)		41.0 (34.2-53.3)	
•I2	12.0 (7.8-14.3)		39.6 (32.9-47.1)	
•I3	9.2 (6.9-10.2)		33.3 (32.3-37.4)	
Ballooning		<0.001		0.001
•B0	10.4 (7.7-14.1)		37.0 (31.0-46.1)	
•B1	13.0 (9.9-18.6)		41.7 (35.1-55.5)	
•B2	12.1 (8.1-16.6)		44.6 (34.4-52.0)	
Fibrosis stage		0.296		0.238
•F0	10.8 (7.6-14.4)		37.1 (31.8-46.1)	
•F1	12.7 (8.1-16.7)		40.5 (31.8-56.9)	
•F2	11.1 (8.3-15.6)		37.2 (32.1-47.9)	
•F3	11.9 (8.7-15.0)		41.0 (35.3-46.8)	
•F4	13.2 (10.2-17.5)		43.2 (36.0-54.6)	
Iron grade		0.781		0.632
•Fe0	11.3 (8.1-16.3)		39.5 (33.2-49.3)	
•Fe1	11.6 (8.6-14.4)		36.4 (30.1-46.0)	
•Fe2	13.5 (8.7-14.0)		41.5 (36.6-45.9)	
•Fe3	13.1 (8.8-18.3)		40.2 (31.0-44.5)	
•Fe4	13.3 (8.7-17.4)		43.5 (34.5-63.5)	
NAFLD activity		<0.001		0.141
•NAFL	11.7 ± 5.6		42.8 ± 19.6	
•NASH	14.4 ± 6.1		46.1 ± 17.6	

Data is expressed as means ± SD or medians with IQR.

NAFL, non-alcoholic fatty liver; NASH, non-alcoholic steatohepatitis.

### 3.3 Pancreatic and liver iron overload

**Figure 5:**
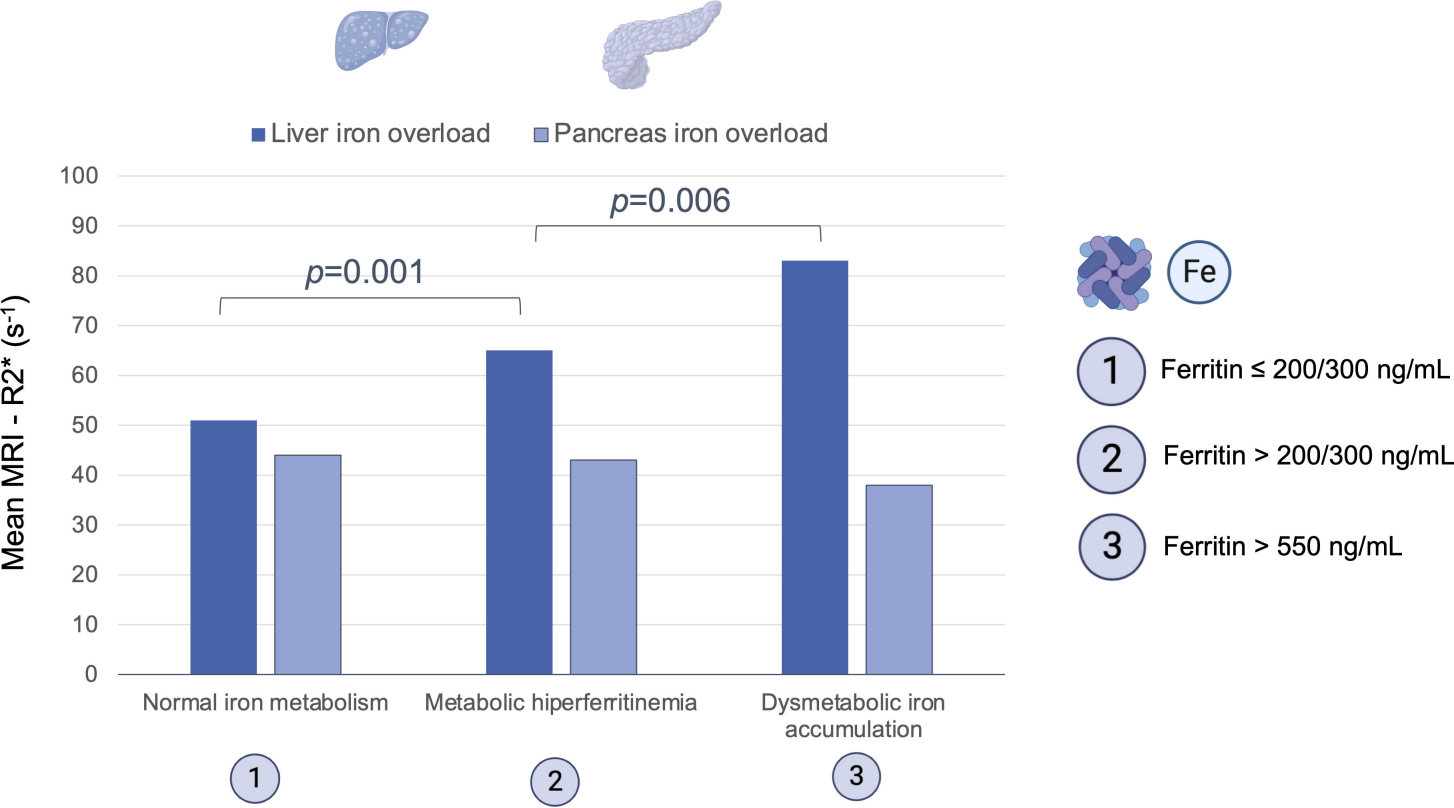
Mean hepatic and pancreatic transverse relaxometry (R2*) across groups of serum ferritin values: normal iron metabolism (≤200 ng/mL women, ≤300 ng/mL men), metabolic hiperferritinemia (>200/300 - 550 ng/mL) and dysmetabolic iron accumulation (>550 ng/mL).

Mean pancreatic and liver MRI-R2* were 44.3 ± 20.6 s^-1^ and 55.9 ± 23.8 s^-1^, respectively. The prevalence of pancreatic iron overload was 45% (n=147), without differences between gender (*p*=0.219). The prevalence of iron overload in the liver was 17% (n=55) and in both pancreas and liver was 9% (**Figure 1**). There was a weak correlation between pancreas R2* and liver R2* (r=0.17, *p*=0.003; **Figure 2**). There was a moderate correlation between pancreas R2* and pancreas PDFF (r=0.64, *p*<0.001). Pancreatic R2* values showed no correlation with hepatic histologic iron deposits grades or digital pathology iron proportionate area (**Figure 3**). Pancreatic iron overload was associated to most metabolic disorders, showing an exponential increase with the number of metabolic traits (**Table 3**). Ferritin levels grading MHF significantly correlated with liver R2* values, being able to differentiate between all severity groups (**Figure 5**). Mean liver R2* among patients with normal iron metabolism was 51 ± 16 s^-1^, in MHF cases it was 65 ± 31 s^-1^, and within dysmetabolic iron accumulation raised to 83 ± 45 s^-1^. For detecting iron metabolism alteration (ferritin >200 in women and >300 in men), the AUC of liver R2* was 0.77 with 95%CI 0.72-0.82 (**Supplementary Material Figure 3**). Correlation between ferritin levels and liver R2* was moderate (r=0.48, *p*<0.001), while no association was found with pancreatic R2* values.

**Table 3:** Distribution of metabolic comorbidities in patients with pancreatic steatosis and iron overload.

Metabolic conditions		Pancreatic PDFF (%)			Pancreatic R2* (s^-1^)		
		< 15.5%	≥ 15.5%	*p* value	< 39 s^-1^	≥ 39 s^-1^	*p* value
Obesity	No	168 (72%)	41 (50%)	<0.001	122 (71%)	87 (59%)	0.028
	Yes	67 (28%)	41 (50%)		49 (29%)	60 (41%)	
DM	No	178 (76%)	48 (59%)	<0.001	133 (78%)	94 (64%)	0.008
	Yes	56 (24%)	34 (41%)		38 (22%)	53 (36%)	
Arterial hypertension	No	158 (67%)	33 (40%)	<0.001	121 (71%)	71 (48%)	<0.001
	Yes	76 (33%)	49 (60%)		50 (29%)	76 (52%)	
Dyslipidemia	No	121 (52%)	29 (35%)	0.010	94 (55%)	57 (39%)	0.004
	Yes	113 (48%)	53 (65%)		77 (45%)	90 (61%)	
Metabolic syndrome	No	179 (76%)	43 (52%)	<0.001	133 (78%)	91 (62%)	0.003
	Yes	55 (24%)	39 (48%)		38 (22%)	56 (38%)	
Age ≥ 55 years	No	132 (56%)	23 (28%)	<0.001	104 (61%)	52 (35%)	<0.001
	Yes	102 (44%)	59 (72%)		67 (39%)	95 (65%)	
Ever smoker	No	177 (76%)	65 (80%)	0.426	131 (77%)	113 (77%)	0.948
	Yes	57 (24%)	17 (20%)		4 0 (23%)	34 (23%)	
ASCVD risk	Low	166 (71%)	33 (40%)	<0.001	129 (75%)	71 (48%)	<0.001
	High	68 (29%)	49 (60%)		42 (25%)	76 (52%)	
Number metabolic traits	0	73 (31%)	10 (12%)	<0.001	60 (35%)	23 (16%)	<0.001
	1	73 (31%)	15 (19%)		49 (29%)	39 (27%)	
	2	41 (18%)	22 (27%)		30 (18%)	33 (23%)	
	3	29 (13%)	18 (23%)		20 (12%)	27 (19%)	
	4	17 (7%)	15 (19%)		11 (6%)	21 (15%)	

Data is expressed as numbers of participants, with percentages in parentheses. Pancreatic PDFF ≥ 15.5% corresponds to moderate-to severe steatosis and R2* ≥ 39 s^-1^ corresponds to iron overload.

ASCVD, atherosclerotic cardiovascular; DM, Type-2 diabetes mellitus.

### 3.4 Cardiovascular risk

**Figure 6:**
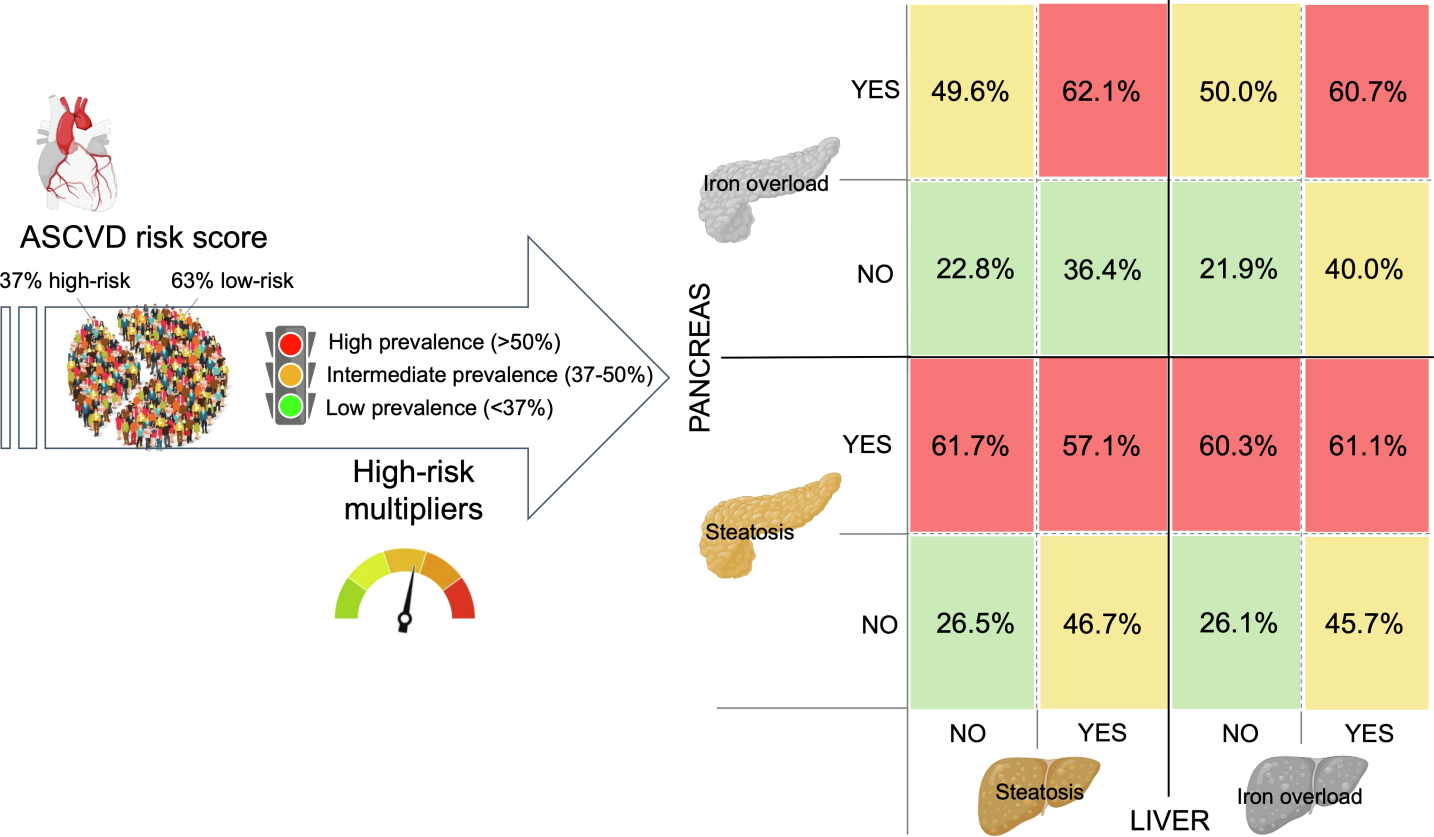
Prevalence (%) of high-risk cardiovascular disease (ASCVD score). The 37% high-risk CVD prevalence in the study sample significantly increased when pancreatic steatosis and iron overload was present. The CVD risk heat map is also stratified by the presence of moderate-to-severe liver steatosis and hepatic iron overload.

The average 10-year risk of CHD was 8.9%, classifying 37% (n=117) of NAFLD patients as intermediate-high risk CVD score (namely high-risk CVD). The calculated 10-year risk of CHD was significantly higher in males (*p*=0.015), obese, ever smokers, patients with dyslipidemia, DM and AHT (*p*<0.001 for all). The prevalence of patients classified as high risk multiplied when pancreatic steatosis and iron overload was present (**Figure 6**). In the multivariate analysis, MRI-determined moderate pancreatic steatosis (OR: 3.15, 95%CI 1.63-6.09; *p*=0.001), and pancreatic iron accumulation (OR: 2.39, 95%CI 1.32-4.37; *p*=0.004) were independently associated with high-risk CVD. Investigating the association of CVD risk with the histological severity of NAFLD, the presence of severe steatosis, hepatocyte balloon degeneration, NASH diagnosis and advanced fibrosis were all retained in the fully adjusted model (**Table 4**).

**Table 4:** Odds ratios for ≥ 7.5% estimated cardiovascular disease risk according to the MRI-metrics and histological severity of NAFLD.

Variable	Univariate analysis		Multivariate analysis †	
	OR (95%CI)	P value	OR (95%CI)	P value
MRI-biomarkers				
Liver PDFF (≥15.5%)	2.03 (1.16-3.55)	0.012	1.69 (0.84-3.42)	0.143
Pancreas PDFF (≥15.5%)	3.74 (2.21-6.33)	<0.001	3.15 (1.63-6.09)	0.001
Liver R2* (≥70 s^-1^)	1.98 (1.09-3.60)	0.023	1.60 (0.75-3.43)	0.223
Pancreas R2* (≥39 s^-1^)	3.34 (2.07-5.38)	<0.001	2.39 (1.32-4.37)	0.004
Histological features				
Moderate-severe steatosis (S2-3)	2.57 (1.59-4.17)	<0.001	2.05 (1.11-3.79)	0.022
Lobular inflammation (I2-3)	0.83 (0.48-1.43)	0.494	0.88 (0.44-1.76)	0.715
Ballooning (B1-2)	2.66 (1.65-4.28)	<0.001	2.23 (1.20-4.13)	0.011
NASH	2.76 (1.70-4.48)	<0.001	2.31 (1.24-4.27)	0.008
Advanced fibrosis (F3-4)	4.59 (2.58-8.17)	<0.001	3.27 (1.63-6.55)	0.001
Iron deposits (Fe1-4)	1.71 (0.93-3.12)	0.081	1.05 (0.50-2.45)	0.805

†Adjusted by age, gender, tobacco consumption and dyslipidemia.

## 4 Discussion

This is a prospective study in a large well-characterized cohort of NAFLD patients that investigated the relationship between the liver and pancreas to assess cardiometabolic risk and iron metabolism disturbances. The key findings of the current investigation are as follows. Pancreatic steatosis and iron overload are common in NAFLD patients and both conditions increase with the number of metabolic traits. MRI-PDFF and R2* values have a weak correlation between liver and pancreas, pointing out organ-independent disorders of fatty acids deposition and dysmetabolic iron overload. Patients with moderate-to severe fatty pancreas have higher risk of NASH, probably associated to insulin resistance. Hepatic iron content determined by MRI correlates with serum levels of ferritin and MHF severity. Finally, patients with high pancreatic PDFF and R2* values are independently associated with increased CVD risk.

The management of NAFLD is based on the non-invasive risk stratification of advanced fibrosis (4). Several cohort studies have suggested that NAFLD-related mortality is mainly due to CVD (3). In Europe, cardiovascular and liver diseases are the two leading causes of years of working life lost (22). Our reported prevalence of high-risk CVD in Spain is similar to studies from Asia but lower than in the USA (14, 23). NAFLD is just one facet of a systemic disease with substantially increased cardiovascular morbidity, but the extent to which the liver injury independently drives CVD is still unclear. For instance, non-invasive tests used to identify advanced liver fibrosis have limited performance in predicting extra-hepatic outcomes (24). In the present study, we have shown that pancreatic steatosis and iron overload are independent factors associated with a high CVD risk. This strong association is critical, as ASCVD score can accurately predict the new onset CHD and overall mortality in NAFLD patients (14, 25). Possible mechanisms linking pancreatic fat with CVD include its involvement in the pathogenesis of DM and incident metabolic syndrome, which in turn are related with increased risk of atherosclerosis (26). Pancreatic steatosis is also independently associated with increased aortic intima-media thickness and epicardial adipose tissue (27). Iron overload in the pancreas causes death of acinar cells and exocrine disfunction which is associated with higher incidence of CV events (6, 28). This mounting evidence can be considered a warning sign for physicians to further risk-stratify NAFLD by classifying disease severity in extra-hepatic organs. Evaluating the pancreas can help to identify the patients that will benefit most from early intervention to prevent CHD events and therefore improve outcomes in NAFLD.

Mean pancreatic PDFF values obtained in our study (12.6%) are in line with other NAFLD cohort studies (**Supplementary Material Table 1**). Pancreatic steatosis has been associated with insulin resistance, DM, and obesity, which are common risk factors for NASH (6, 7). Our findings of increased pancreatic fat accumulation in NAFLD patients with DM and non-diabetic patients with elevated markers of insulin resistance, offers additional explanation for the link between pancreatic steatosis and NASH. The magnitude of association (OR, 95%CI) between pancreatic steatosis and DM was very similar to the one reported in a recent meta-analysis (7). Previous investigations support our data as it has been shown that the number of metabolic traits and moderate glycemic control may increase the risk of NASH (12, 29). Furthermore, significant differences in the correlation between adipose tissue dysfunction (VAI and BMI) and MRI-PDFF of the liver and pancreas point to a possible organ-independent deposition of fatty acids (8, 10). These different pathological pathways justify the consistent results not showing a connection between pancreatic disease and liver fibrosis severity (30).

A prevalence of pancreatic iron overload x2.5 times higher than liver iron accumulation was found in our series. Hepatic and extra-hepatic organ iron deposition in NAFLD has been scarcely investigated (9). Our investigation fills this research gap and determines the correlation between serum levels of ferritin and hepatic iron content in patients with MHF and NAFLD. A similar correlation rate was described by França et al, in patients with other chronic liver diseases (31). Liver R2* was able to non-invasively diagnose MHF and discriminate dysmetabolic iron overload groups with clear cut-offs. Identifying iron induced organ damage is important as MHF is associated with an increased risk of cardiometabolic diseases (9). We also raise the hypothesis that pancreatic iron level might be implicated in CVD risk in an exponential manner with liver iron deposits (**Figure 6**). This findings are consistent with previous studies suggesting that MRI-determined liver R2* can predict adverse outcomes and do not correlate with pancreatic R2* (31, 32).

Our study has some limitations. First, the cross-sectional design with absence of follow-up restricts the observed results to correlations, not possibly linked with incident events. Future studies should validate these results in longitudinal investigations evaluating the implication of pancreatic disease in the liver injury. Second, histopathological proof of fat and iron accumulation in the pancreas was not obtained as biopsies in this organ are precluded for ethical constraints. Histological information about pancreatic fat accumulation is based upon studies that analyses samples taken during pancreatic surgery (33). Histological findings reveal that adipocytes predominantly accumulate interlobularly more than intra-cellular. The heterogeneity in the distribution of pancreatic steatosis in our MR images also suggest that extracellular inter-lobular adipocyte infiltration is the main component, although intra-cellular lipid accumulation can also be involved (**Supplementary Material Figure 4**) (26). Third, homeostatic model of insulin resistance (HOMA-IR) was not possibly calculated. Glucose and HbA1c are suboptimal surrogated markers of insulin resistance in comparison with HOMA (29). There are also strengths in this study. Our cohort has a large number of NAFLD patients, from different centers, all with paired biopsy and MRI as reference diagnostic techniques. MRI protocol obtained a multiecho chemical shift-encoded gradient echo sequence considering only the water contribution to the R2* measurements, controlling the confounding factor of fat (5, 34). Image definition of pancreatic and liver disease are based on meta-analysis and consensus statements (7, 9, 15, 16). The prospective study includes a well-characterized population that captures real-world data from clinicians´ evaluation of patients with NAFLD. To reduce interobserver bias in the histopathologic reading, liver biopsies were evaluated in a centralized single institution and computational digital pathology was also applied. There was a short time interval between biopsy and MRI (average of 19 days). Finally, ASCVD score was assessed as a relevant outcome to depict high-risk multipliers like pancreatic steatosis and iron overload that might improve the accuracy to predict adverse events in NAFLD patients (3, 14).

In summary, pancreatic steatosis and iron overload is common in NAFLD. Abdominal MRI performed for the study of liver diseases should include the evaluation of the pancreas as its disease reveals a higher risk of CVD. Pancreatic steatosis and iron overload should factor into clinical decision-making and prognostication of patients with NAFLD.

## Data availability statement

The original contributions presented in the study are included in the article/**Supplementary Material**. Further inquiries can be directed to the corresponding author.

## Ethics statement

The studies involving human participants were reviewed and approved by Clinic University Hospital, INCLIVA Health Research Institute (2016/209 ethics committee registry); La Fe Health Research Institute (2017/0031/PI ethics committee registry); Hospital Arnau de Vilanova (13/2019 ethics committee registry); and Research Foundation of the General Hospital of Valencia, Spain (106/2021 ethics committee registry). The patients/participants provided their written informed consent to participate in this study. Written informed consent was obtained from the individual(s) for the publication of any potentially identifiable images or data included in this article.

## Author contributions

DM-A, AT-E, AA-B and LM-B: study concept and design. DM-A, CB-S, AC, EC, VM-M, SB, VA, ML, CM, and DE-G: acquisition data. MF, CA-C, AS-M, MB, JP-R, VP and AF: histological analysis. DM-A, CB-S, AT-E, AJ-P, AP-G, AA-B and LM-B: image analysis. DM-A, AJ-P, AA-B, and LM-B: statistical analysis and interpretation of data. DM-A, and LM-B: drafting of manuscript. AT-E, CB-S, AC, EC, VM-M, MF, CA-C, AS-M, MB, AJ-P, AP-G, SB, JP-R, VP, AF, VA, ML, CM, DE-G, IB-R, and AA-B: critical revision of the manuscript. All authors approved the final version of the article: DM-A, AT-E, CB-S, AC, EC, VM-M, MF, CA-C, AS-M, MB, AJ-P, AP-G, SB, JP-R, VP, AF, VA, ML, CM, DE-G, IB-R, AA-B, and LM-B. All authors contributed to the article and approved the submitted version.
