# Supplementary material for: Pancreatic steatosis and iron overload increases cardiovascular risk in non-alcoholic fatty liver disease
Source: Front Endocrinol (Lausanne). 2023 Aug 3;14:1213441. doi: 10.3389/fendo.2023.1213441 (PMC10436077; doi:10.3389/fendo.2023.1213441)
Supplement: Supplementary file 1 [file DataSheet_1.docx]

Supplementary Material

**Pancreatic Steatosis and Iron Overload Increase Cardiovascular Risk in Non-alcoholic Fatty Liver Disease**

# Supplementary Figures and Tables

**
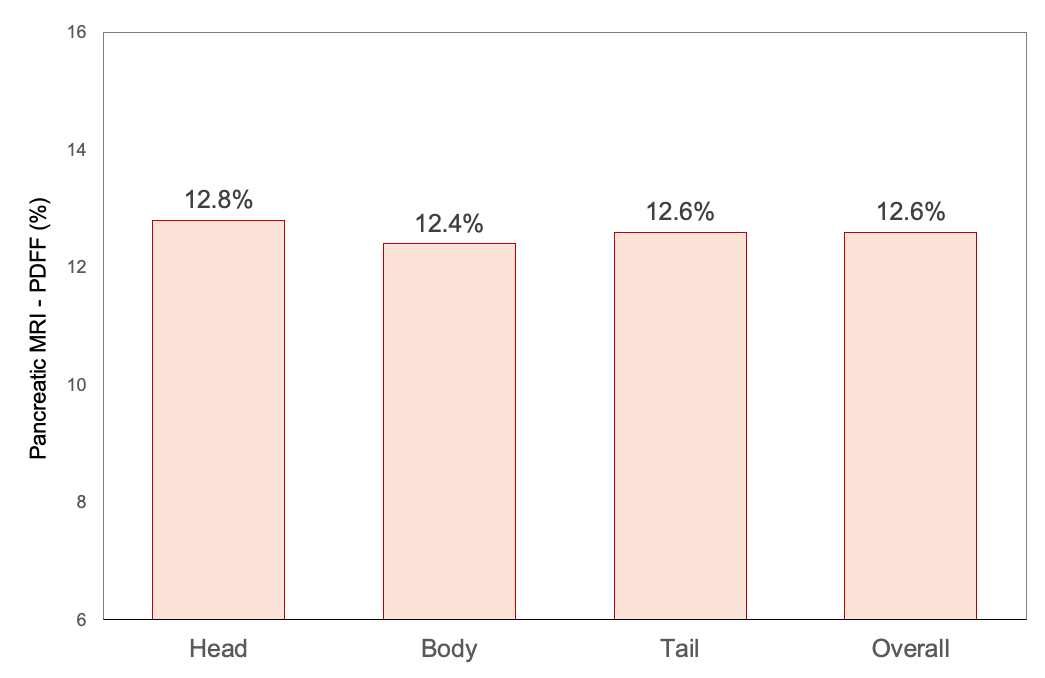
**

**Supplementary Figure 1.** Magnetic resonance image (MRI) determined pancreatic fat across regions of the pancreas. Mean proton density fat-fraction (PDFF) is shown for the head, body, and tail of the pancreas. Overall mean MRI-PDFF was calculated as the mean of the three regions. Paired t-tests showed no statistical difference in MRI-PDFF between regions of the pancreas: head *vs.* body *p*=0.52; head *vs*. tail *p*=0.81; and body *vs* tail *p*=0.71.


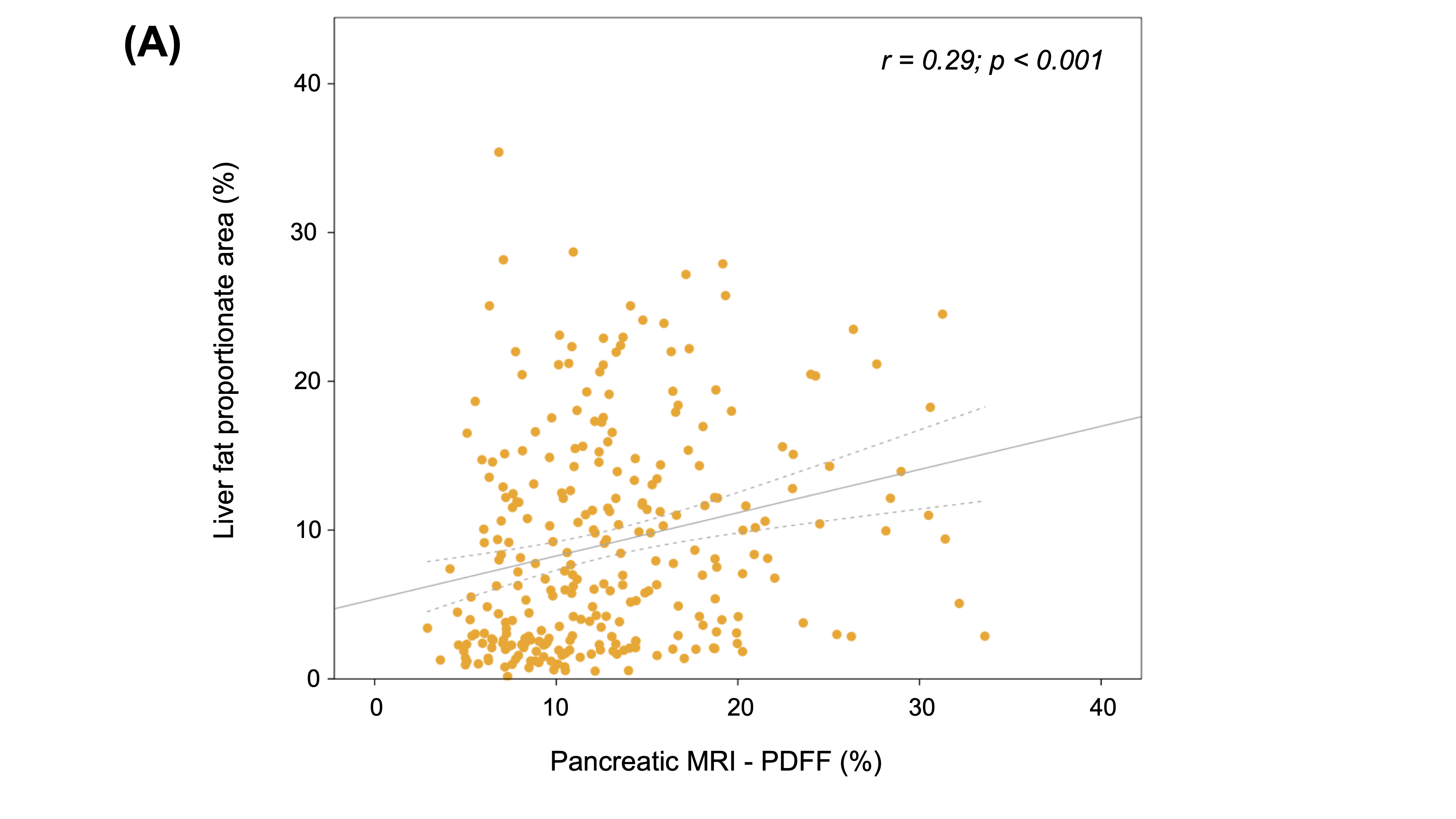


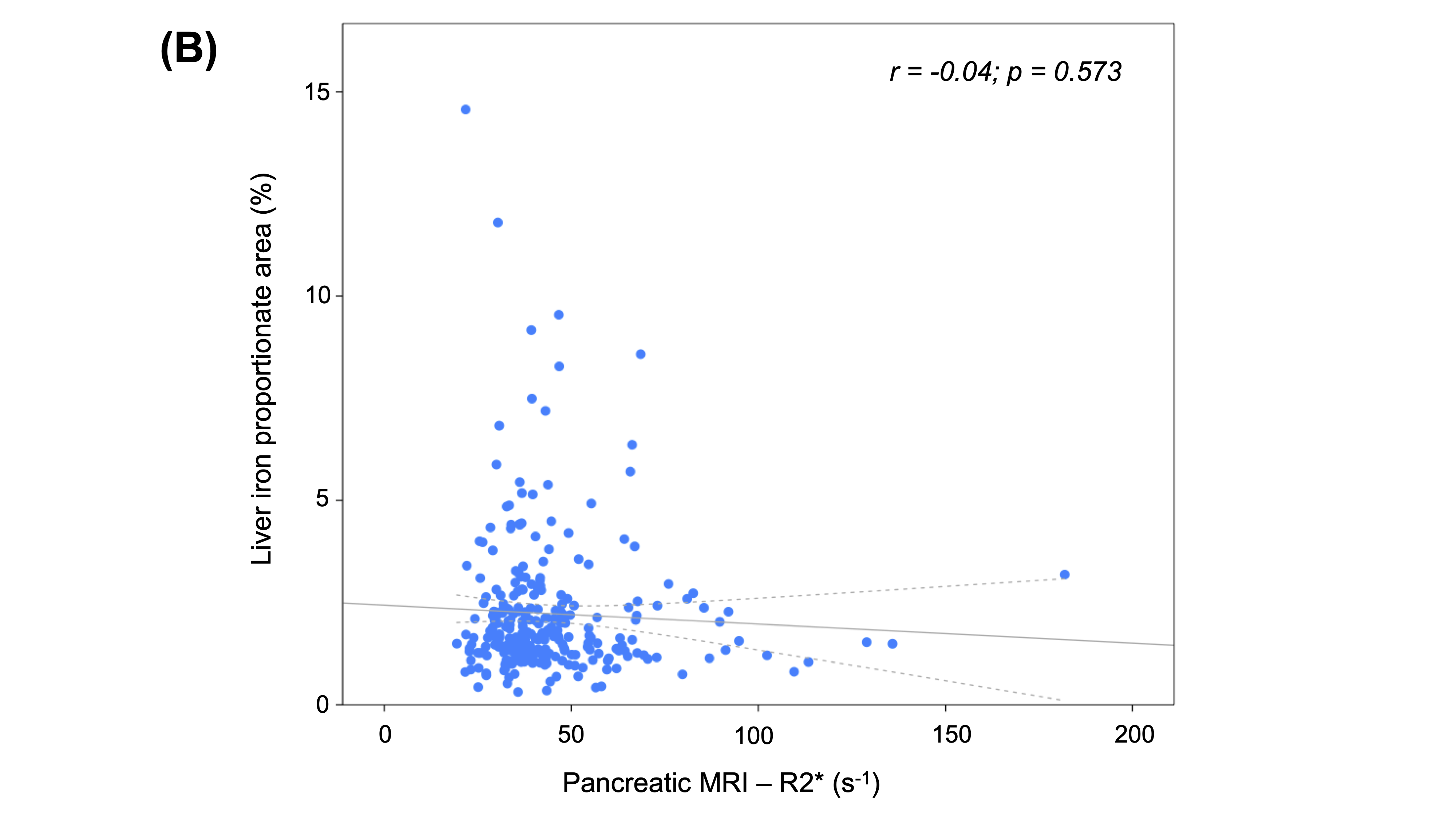


**Supplementary Figure 2.** Scatterplots of MRI parameters and digital pathology. **(A)** Proton density fat fraction (PDFF) versus fat ratio, and **(B)** transverse relaxometry (R2*) versus iron ratio. The gray line represents the linear regression fit and the dotted lines the 95% confidence interval.

**Supplementary Figure 3.** Receiver operating characteristic curve for MRI-R2* for discriminating the presence of iron metabolism alteration.


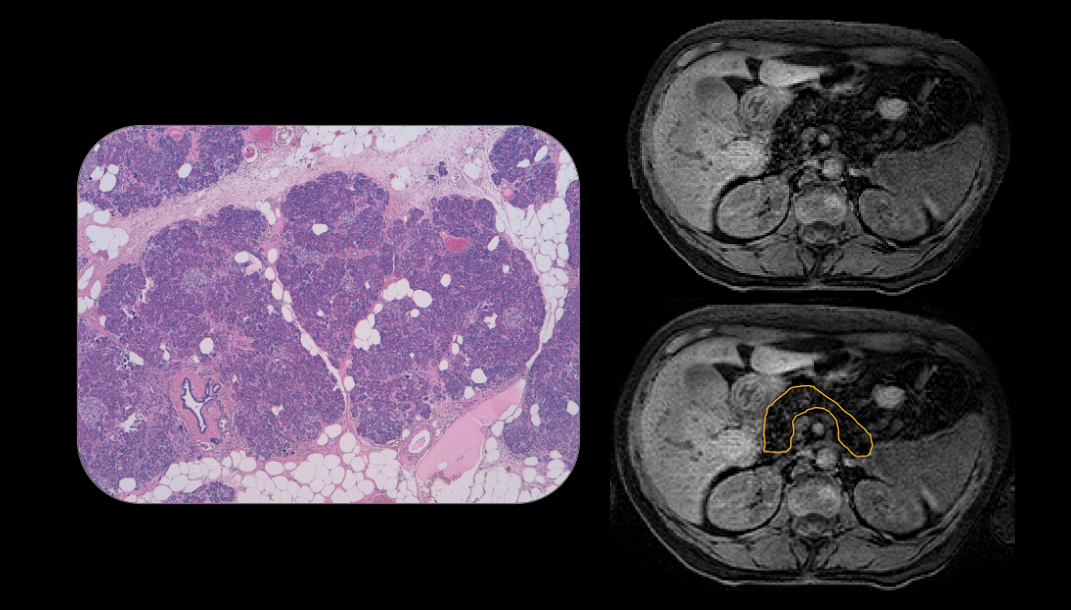


**Supplementary Figure 4.** Hypothesis of pancreatic fat infiltration. Pancreatic histology with fatty infiltration showing that adipocytes predominantly accumulate interlobularly, and the remaining pancreas lobules resemble islets surrounded by a fatty lake. MR image out-of-phase second echo (1.6 msec) gradient-recalled echo sequence with proton density fat fraction quantification (PDFF: 30%). Note the heterogeneity in signal intensity reduction of the whole pancreas representing fatty infiltration. The heterogeneity in the distribution of pancreatic steatosis suggest that extracellular inter-lobular adipocyte infiltration is the main component The pancreas is outlined by the yellow line in the right bottom image. Histology and MR image do not belong to the same patient.

**Supplementary Table 1.** Summary of studies evaluating fat and iron deposits in the pancreas with MRI methods in NAFLD population.

| **Study** | **Population** | **Methodology** | **Pancreatic results** |
| --- | --- | --- | --- |
| Idilman IS 2021 [1] | - n=86 bariatric surgery - n=49 NAFLD - Biopsy (54% NASH) - USA | - MRI-PDFF - 90-day interval MRI-biopsy - Retrospective - Single center | - Mean PDFF: 12.7% - HOMA-IR: r=0.23 - VAT: r=0.29 - No correlation with liver histology - Liver PDFF: r=0.43 |
| Boga S 2020 [2] | - n=57 NAFLD - Biopsy (70% NASH) - Turkey | - MRI-PDFF - 25-day interval - Single center | - Mean PDFF: 10.1% - HOMA-IR: r=0.35 - No correlation with NASH or liver PDFF |
| Shur J 2020 [3] | - n=105 haematological disorders - n=8 controls - Canada | - MRI-PDFF - MRI-R2* - Retrospective - Single center | - Mean PDFF: 17.3% - Mean R2*: 119 s^-1.^ - Liver R2*: r=0.02 - Liver R2*: r=0.20 |
| Kato S 2019 [4] | - n=159 NAFLD - Biopsy - Japan | - MRI-PDFF - 6-month interval - Retrospective - Single center | - No correlation with HOMA-IR - No correlation with NASH or liver PDFF |
| Vieira J 2019 [5] | - n=46 CLD - Biopsy (59% NAFLD) - Portugal | - MRI-PDFF - Single center | - Mean PDFF: 11.5% - BMI: r=0.41 - Steatosis grade: r=0.57 - Liver PDFF: r=0.54 |
| França M 2018 [6] | - n=56 CLD - Biopsy (16% NAFLD) - Portugal | - MRI-R2* - 2-day interval - Single center | - Mean R2*: 34 s^-1.^ - Ferritin: r=0.30 - No correlation with histologic iron grades - Liver R2*: r=0.22 |
| Idilman IS 2015 [7] | - n=41 NAFLD - Biopsy (70% NASH) - Turkey | - MRI-PDFF - 16-day interval - Single center | - Mean PDFF: 5.7% - BMI: r=0.32 - No correlation with HOMA-IR or liver fibrosis (histology) - Liver PDFF: r=0.10 |
| Patel NS 2013 [8] | - n=43 NAFLD - Biopsy (49% NASH) - USA | - MRI-PDFF - 43-day interval - Prospective, cross-sectional - Single center | - Mean PDFF: 8.5% - BMI: R^2^=0.02 - Histologic correlation with steatosis grade and NASH (*P*=0.03) |

Abbreviations: Body Mass Index, BMI; Chronic liver disease, CLD; Magnetic Resonance Imaging, MRI; non-alcoholic fatty liver disease, NAFLD; non-alcoholic steatohepatitis, NASH; Proton Density Fat Fraction, PDFF; Insulin resistance, IR.

**References:**

1. Idilman IS, Low HM, Gidener T, et al. Association between Visceral Adipose Tissue and Non-Alcoholic Steatohepatitis Histology in Patients with Known or Suspected Non-Alcoholic Fatty Liver Disease. *J Clin Med*. 2021; 10(12):2565.
2. Boga S, Koksal AR, Sen İ, et al. Liver and pancreas: 'Castor and Pollux' regarding the relationship between hepatic steatosis and pancreas exocrine insufficiency. *Pancreatology*. 2020; 20(5):880-886.
3. Shur J, Kannengiesser SAR, Menezes R, Ward R, Kuo K, Jhaveri K. Glucose dysregulation in patients with iron overload: is there a relationship with quantitative pancreas and liver iron and fat content measured by MRI?. *Eur Radiol*. 2020; 30(3):1616-1623.
4. Kato S, Iwasaki A, Kurita Y, et al. Three-dimensional analysis of pancreatic fat by fat-water magnetic resonance imaging provides detailed characterization of pancreatic steatosis with improved reproducibility. *PLoS One*. 2019; 14(12):e0224921.
5. Vieira J, Amorim J, Martí-Bonmatí L, Alberich-Bayarri Á, França M. Quantifying steatosis in the liver and pancreas with MRI in patient with chronic liver disease. Cuantificación por resonancia magnética de esteatosis hepática y pancreática en hepatopatía crónica. *Radiologia (Engl Ed).* 2020; 62(3):222-228.
6. França M, Martí-Bonmatí L, Porto G, et al. Tissue iron quantification in chronic liver diseases using MRI shows a relationship between iron accumulation in liver, spleen, and bone marrow. *Clin Radiol*. 2018; 73(2):215.e1-215.e9.
7. Idilman IS, Tuzun A, Savas B, et al. Quantification of liver, pancreas, kidney, and vertebral body MRI-PDFF in non-alcoholic fatty liver disease. *Abdom Imaging*. 2015; 40(6):1512-1519.
8. Patel NS, Peterson MR, Lin GY, et al. Insulin Resistance Increases MRI-Estimated Pancreatic Fat in Nonalcoholic Fatty Liver Disease and Normal Controls. *Gastroenterol Res Pract*. 2013; 2013:498296.

**Supplementary Table 2.** Histopathologic characteristic distribution in the study sample.

| Histological feature | Patients |
| --- | --- |
| Moderate-severe steatosis (n, %) | 124 (38.3%) |
| Lobular inflammation (n, %) |  |
| - No foci | 95 (29.3%) |
| - < 2 foci per 200X field | 141 (43.5%) |
| - 2-4 foci per 200X field | 73 (22.5%) |
| - > 4 foci per 200X field | 15 (4.7%) |
| Ballooning grade (n, %) |  |
| - None | 174 (53.7%) |
| - Few balloon cells | 106 (32.7%) |
| - Many cells/prominent ballooning | 44 (13.6%) |
| Fibrosis stage (n, %) |  |
| - F0: none | 108 (33.3%) |
| - F1: perisinusoidal or periportal | 68 (21.0%) |
| - F2: perisinusoidal and portal/periportal | 70 (21.6%) |
| - F3: bridging fibrosis | 47 (14.5%) |
| - F4: cirrhosis | 31 (9.6%) |
| Iron overload grade (n, %) |  |
| - Granules absent/barely discernible at x400 | 235 (81.3%) |
| - Granules barely discernible at x250 and easily confirmed at x250 | 16 (5.5%) |
| - Discrete granules resolved at x100 | 14 (4.8%) |
| - Discrete granules resolved at x25 | 16 (5.5%) |
| - Masses visible at x10, or naked eye | 8 (2.9%) |

Note: data is expressed as numbers of participants, with percentages in parentheses.

**Supplementary Table 3.** Distribution of surrogated markers of insulin resistance in non-diabetic cohort (n=230)

|  | None-low liver steatosis | Moderate-to severe liver steatosis | *p* value | None-low pancreatic steatosis | Moderate-to severe pancreatic steatosis | *p* value |
| --- | --- | --- | --- | --- | --- | --- |
| Fasting glucose (mg/dL) | 92 [85-102] | 102 [91-110] | 0.002 | 92 [85-102] | 99 [88-112] | 0.013 |
| HbA1c (%) | 5.4 [5.2-5.8] | 5.8 [5.5-6.0] | 0.022 | 5.4 [5.2-5.8] | 5.8 [5.6-6.2] | 0.001 |

Note: data is expressed as median, with interquartile range in parentheses. Moderate-to severe steatosis was defined as PDFF ≥15.5%. Differences between groups were evaluated using Mann-Whitney U test.
